# Socioeconomic inequality in tobacco expenditure in Iran: a cross-sectional analysis at national and subnational levels

**DOI:** 10.1186/s12889-020-09144-z

**Published:** 2020-06-29

**Authors:** Satar Rezaei, Mohammad Habibullah Pulok, Mohammad Ebrahimi

**Affiliations:** 1 Research Center for Environmental Determinants of Health, Health Institute, Kermanshah University of Medical Sciences, Kermanshah, Iran; 2 Nova Scotia Health Authority, Halifax, Nova Scotia Canada; 3 Kermanshah University of Medical Sciences, Kermanshah, Iran

**Keywords:** Tobacco expenditure, Inequality, Decomposition, Socioeconomic status, Iran

## Abstract

**Background:**

Tobacco expenditure has adverse impacts on expenditure on basic needs and resource allocation of the households. Using data from a nationally representative survey, we measured socioeconomic inequality in tobacco expenditure as the share of household budget (TEHB) and explained its main determinants among Iranian households at the national and sub-national levels.

**Methods:**

This cross-sectional study used data from the Iranian Household Income and Expenditure Survey (IHIES), 2018. We included a total of 7649 households with tobacco expenditure more than zero in the analysis. Province-level data on the Human Development Index (HDI) was obtained from the Institute for Management Research at Radbound University. The concentration curve (CC) and the concentration index (*C*) were used to measure socioeconomic inequality in TEHB at national and sub-national levels. The *C* was decomposed to identify the factors explaining the observed socioeconomic inequality in TEHB.

**Results:**

At the national level, households with at least one smoker spent more than 5% of their budget for tobacco consumption in the last month. Households from the urban areas allocated less of their budgets on tobacco products compared to rural households (4.6% vs. 5.8%). Overall, TEHB was more concentrated among the poorer households (*C* = 0.1423, 95% CI: − 0.1552 to − 0.1301). In other words, the distribution of TEHB was pro-poor in Iran. Pro-poor inequality in TEHB was also found in urban (*C* = − 0.1707, 95% CI: − 0.1998 to − 0.1516) and rural (*C* = − 0.1314, 95% CI: − 0.1474 to − 0.1152) areas. We also found that pro-poor inequalities were higher in Iranian provinces with low HDI. The decomposition results indicate that wealth and education were the main factors contributing to the concentration of TEHB among the poorer households.

**Conclusion:**

This study found that TEHB was disproportionality concentrated among poorer households in Iran. The extent of inequality in TEHB was higher in urban areas and less developed provinces. Designing and implementing tobacco control interventions to decrease the smoking prevalence and increase smoking cessation could protect worse-off households against the financial burden of tobacco spending.

## Background

Tobacco consumption remains as one of the significant public health problems in many countries around the world [1, 2]. It is one of the leading causes of deaths, accounting for more than seven million deaths globally each year [3]. The negative consequences of tobacco consumption extend beyond health to adverse affects on economic spending in households [4–7]. There are economic impacts of tobacco use at the individual, households, and national levels [8]. For example, tobacco smoking accounts for about 15% of the total healthcare expenditure in developed countries [9]. In Iran, about 0.26% of gross domestic product (GDP) was attributed to the consumption of tobacco in 2014 [10].

Tobacco expenditure has direct and indirect effects of households’ budget [4–6, 11]. The direct effect is defined as “crowding out” effect that decreases the share of expenditure on other necessary goods and services. Evidence from 40 low- and-middle income countries shows that spending on tobacco lowers households’ expenditure on education and healthcare [12]. For example, money spent on tobacco significantly reduced spending on healthcare, insurance, education etc. among the rural households in China [5]. The indirect effect could be an increase in healthcare spending of the household budget due to harmful effects of smoking, loss of income due to illness and productivity loss [8].

Generally, households’ share of the budget on purchasing tobacco-related good ranges from 1 and 10% in different countries [13]. However, tobacco expenditure as the proportion of the households’ budget (TEHB) affects poorer and vulnerable populations to a greater extent, both in developed and developing countries [12, 14]. Households with low socioeconomic status (SES) spent a higher proportion of their budgets on tobacco consumption compared to the households with better SES [4, 6, 15]. For instance, low-income Turkish households with smoker members spent about 8% of monthly budgets on smoking [4]. In New Zealand, if poorer households with at least a smoker become smoker-free, about 14% of the non-housing resources in those households could be available to buy other goods and services [14]. Evidence from China showed a negative association between having at least a smoker in the household and money available for basic needs [15]. In Bangladesh, low-income households with tobacco consumers spent less money on education, housing, transportation, communication, clothing, and energy compared to households with no consumers of tobacco [8]. In addition, tobacco expenditure had a negative impact on consumption of food grains among poorer households with smokers in India [16].

Despite increasing efforts to control the consumption of various tobacco products, the prevalence of smoking remains high in Iran (23.4% for men and 1.4% for women) [17]. There exist several Iranian studies on the costs of smoking, total death attributable to tobacco smoking, and the association between smoking and length of stay in hospital [10, 18, 19]. Few studies from Iran and other developing countries also investigated socioeconomic inequalities tobacco consumption [20–24]. Understanding inequalities in tobacco use and TEHB would allow us to understand the socioeconomic pattern of this problem comprehensively. However, no study has yet examined inequality in TEHB by socioeconomic status in Iran. Using nationally representative data household data, this study aimed to fill this gap in the literature by quantifying the degree of socioeconomic inequality in TEHB and explaining the drivers of this inequality in Iran. We also conducted disaggregated analyses at rural/urban and regional levels based on development status. The results of this study would help designing and implementing policies to decrease the smoking prevalence and increase cessation of smoking. These policies could be helpful to lessen inequalities in tobacco consumption and its expenditures in Iran in the long run.

## Methods

### Study setting

Iran is a developing country, located in the Eastern Mediterranean Region (EMR). According to the 2016 census data, the population of Iran was about 80 million, living across 31 provinces. The proportion of households with tobacco expenditure more than zero in the last month was about 20% (Table S1 in the supplementary file). These proportions for provinces based on their Human Development Index (HDI) were 18.7%, 22.9, and 18.8% (high, middle, and low, correspondingly). Direct taxation rate on Iranian and non-Iranian cigarette is determined at 10 and 40% levels, respectively.

### Data and variables

Data of this study were drawn from the Household Income and Expenditure Survey (HIES) of Iran, 2018. The survey was conducted by the Iranian Statistical Centre (ISC) (https://www.amar.org.ir/english/Statistics-by-Topic/Household-Expenditure-and-Income#287685-definitions%2D%2Dconcepts). Data in this survey were collected from the household heads in a face-to-face interview using a comprehensive questionnaire. The ISC employed a three-stage cluster sampling method to select samples from both rural and urban areas of Iran. Data on sociodemographic characteristics of the households (e.g., age, gender, and education of household head), household’s income and expenditures (e.g., food, clothing, transport, communication, healthcare, food, tobacco etc.) in the past month before the survey were collected from the households included in the survey. Therefore, the unit of analysis in this study is household. All information on income and expenditure of households were reported in Iranian Rials (IRR). The survey collected data from 38,859 households, but we included 7649 households with tobacco expenditure more than zero in the last month in the analysis.

The outcome variable of our study is the tobacco expenditure as the share of the household budget (TEHB) in the last month before the survey. Tobacco expenditure includes the cost of cigarette and other smoked tobacco products consumed by the members of the household. We selected the explanatory variables of this study following the existing literature in this area of research [23, 25, 26]. Using the available information from the IHIES, we included the age of the household head, gender of the household head, educational status of the household head, household size, proportion of males in the household, the wealth index of the household as the proxy for socioeconomic status, and living areas (urban/rural). We also included the development status of provinces based on their HDI score (low, middle, and high) as the determinant of TEHB. We obtained province-level data on HDI from the Institute for Management Research, Radbud University [27].

We applied the Principle Component Analysis (PCA) method to construct the wealth index for each household [28]. Several characteristics of the households (e.g. number of rooms, type of house ownership, house size per square meter) and durable assets of the households (e.g. car, TV colour, internet, computer/laptop, cell phone, freezer, dishwasher, microwave, vacuum cleaner, motorcycle and bicycle) were included in the PCA to calculate the wealth score. Households were classified into five socioeconomic status (SES) groups from the poorest (first quintile) to the richest (fifth quintile) according to their wealth score.

### Statistical analysis

This study used the concentration curve (CC) and the concentration index (*C*) to assess and measure socioeconomic inequality in TEHB [29]. The CC plots the cumulative proportion of the households ranked by wealth index on the x-axis, against the cumulative percentage of TEHB on the y-axis. If TEHB is equally distributed across the households, the CC will be a 45-degree line, known as the line of equality. If the CC of TEHB lies under the line of equality, it indicates that the TEHB is more concentrated among richer and vice versa. The C could be estimated as twice the area between CC and the line of equality. The *C* takes value − 1 to + 1 where a positive sign suggests that the TEHB is more prevalent among the households with higher SES and vice versa. A zero value of the C implies no inequality in the outcome variable. We used the following formula to calculate the C:

1 $$ 2{\sigma}_r^2\left(\frac{y_i}{\mu}\right)=\upalpha +\upvarphi {r}_i+{\varepsilon}_i $$

Where *μ* is the mean of the dependent variable (i.e., TEHB) for the whole sample; *y*_*i*_ refers to the outcome variable (in this study TEHB) for the household *i*; and *r*_*i*_ is the fractional rank of the household *i* in the SES distribution; ($$ {r}_i=\raisebox{1ex}{$i$}\!\left/ \!\raisebox{-1ex}{$n$}\right. $$, where n is the sample size), and $$ 2{\sigma}_r^2 $$ stands for the variance of the fractional rank. The ordinary least squares (OLS) estimate of φ is the *C* [30].

We applied the decomposition approach to estimate the contribution of explanatory variables to SES-related inequality in TEHB [31]. In the decomposition analysis, a set of *k* explanatory factors ,*x*_*k*_, were regressed on the outcome variable, *y*, in a linear regression model as follows:

2 $$ y=\alpha +\sum \limits_k{\beta}_k{x}_k+\varepsilon $$

Wagstaff et al. [31] showed that the *C* can be decomposed to its determinants using the following formula:

3 $$ C=\sum \limits_k\left(\frac{\beta_k{\overline{x}}_k}{\mu}\right){RC}_k+\frac{G{C}_{\varepsilon }}{\mu } $$

Where ${\overline{x}}_k$ and *C*_*k*_ are the mean and the *C* of the explanatory variables. If the *C* of an explanatory variable is negative, it indicates that variable is more concentrated among the poor and vice versa. $\frac{\beta_k{\overline{x}}_k}{\mu }$ is the elasticity of TEHB with respect to the explanatory variables *x*_*k*_. Elasticity measures the amount of change in TEHB related to one-unit change in the explanatory variable. If the elasticity for an explanatory variable is positive, an increase in that variable increases TEHB and vice versa. The last term $\frac{G{C}_{\varepsilon }}{\mu }$ is the generalized concentration index for the error term.

The absolute contribution of each factor is a function of the elasticity of TEHB with respect to that factor and the *C* of this factor. A large elasticity or a large *C* or both results in a large contribution to the observed inequality in TEHB. In the decomposition approach, the absolute contribution indicates how much of the relationship between the wealth index of the household and TEHB is explained by the variation in a specific explanatory variable across the socioeconomic distribution. In addition, the relative contribution of each determinant was calculated by dividing the absolute contribution for that determinant by the *C* and then multiplying by 100. For example, when the relative contribution of an explanatory variable (i.e., education status of head of household) i equals to − 10%, an equal distribution of education status of head of household among the socioeconomic groups would cause a 10% increment in socioeconomic inequality in the outcome variable. Stata version 14.2 (Stata Corp., College Station, TX, USA) was used to perform all the analyses and *p*-value less than 0.05 was considered statistically significant. The geographical map was depicted by ArcGIS software Version 10.6.1.

## Results

### Descriptive statistics

**Fig. 1:**
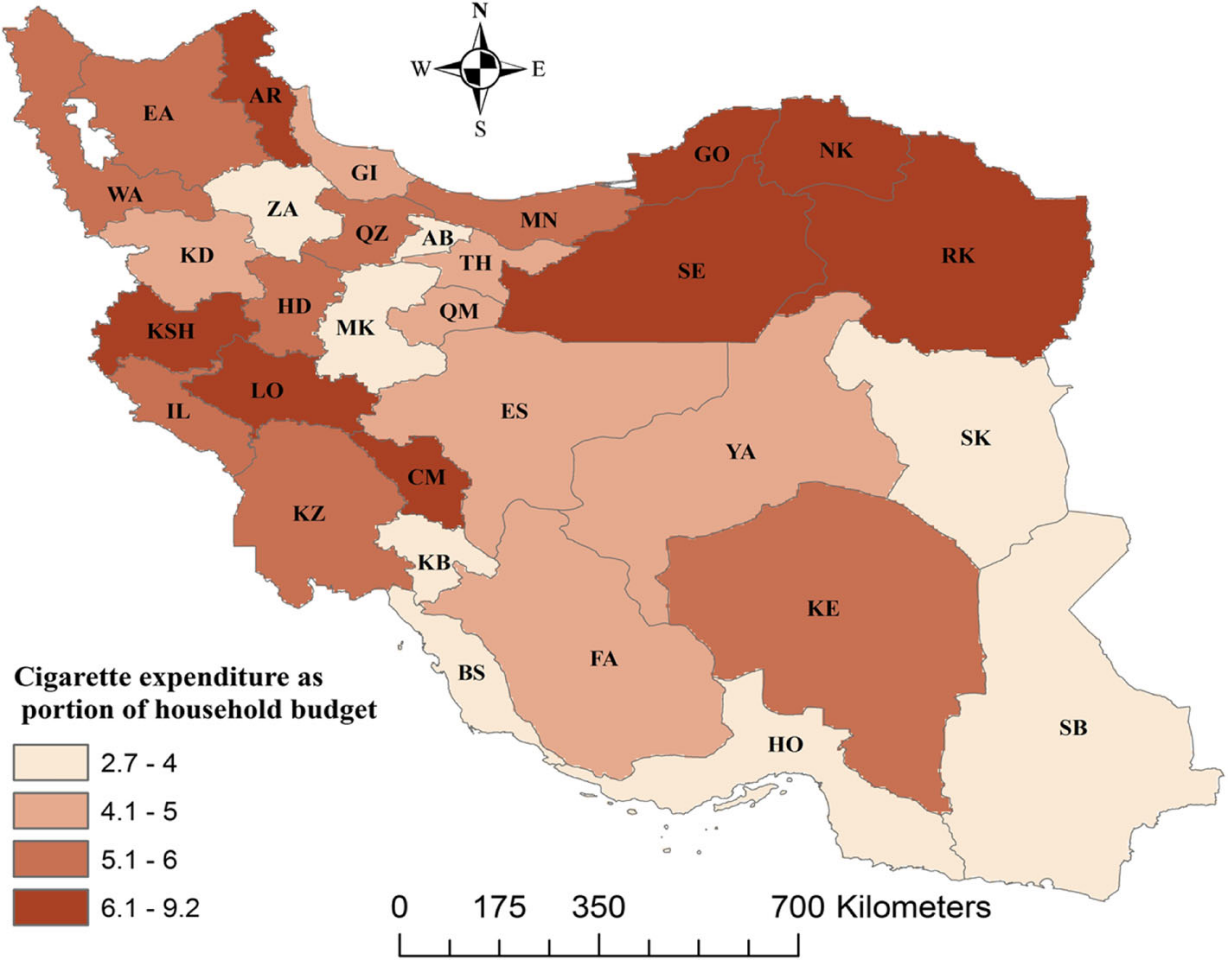
Portion of household budget spent on tobacco product over the last month across provinces in Iran, 2018. **Note:** TH, Tehran; MK, Markazi; GI, Gilan; MN, Mazandaran; EA, East Azerbaijan; WA, West Azerbaijan; KS, Kermanshah; KZ, Khuzestan; FA, Fars; KE, Kerman; RK, Razavi Khorasan; ES, Esfahan; SB, Sistan and Baluchestan; KD, Kurdistan; HD, Hamadan; CM, Chahar Mahall and Bakhtiari; LO, Lorestan; IL, Ilam; KB, Kohgiluyeh and Buyer-Ahmad; BS, Bushehr; ZA, Zanjan; SM, Semnan; YA, Yazd; HG, Hormozgan; AR, Ardebil; QM, Qom; QZ, Qazvin; GO, Golestan; NK, North Khorasan; SK, South Khorasan; AB, Alborz (developed by the authors using ArcGIS Desktop version 10.6.1)

Descriptive characteristics of the households included in the study are reported in Table 1. About 95% of the household heads were men, and 53.4% were from rural areas. The average age (standard deviation) of the household heads was 49.9 year (6.3). On average, the proportion of households’ budget spent on tobacco consumption in the past month was 5.2, 4.6, and 5.8% for the entire sample, urban, and rural households, respectively. Figure 1 shows a significant variation in TEHB across 31 provinces of Iran. For example, 9.2% of total cost of household was related to the tobacco consumption in North Khorasan, while it was about 2.7% in Kohgiluyeh Buyer Ahmad.

**Table 1:** Descriptive characteristics of households included in the analysis, 2018

Variables	Mean [32]	n (%)	Tobacco expenditure as % of household budget		p-value
			%	SD	
*Demographic variables*					
Age of household head (years)	49.9 (13.6)		5.2	5.2	0 < 0.001
Sex of household head					0 < 0.001
Male		7254 (94.8)	5.1	5.0	
Female		394 (5.2)	6.6	8.7	
Proportion of males in the household		55.2	5.2	5.2	0.017
*Socioeconomic variables*					
Household size					0.991
Less than 4		3643 (47.6)	5.5	5.5	
4 and above		4006 (52.4)	5.0	5.1	
Education status of household head					0 < 0.001
Illiterate		1585 (20.7)	6.8	6.9	
Literate		6064 (79.3)	4.8	4.6	
Economic status of households					0 < 0.001
Poorest		1422 (18.6)	7.6	7.5	
Poor		1695 (22.2)	5.8	5.2	
Middle		1652 (21.6)	5.1	4.7	
Rich		1511 (19.7)	4.0	3.8	
Richest		1369 (17.9)	3.6	3.3	
*Ecological variables*					
Geographical area					0 < 0.001
Urban		3592 (47.0)	4.6	4.9	
Rural		4057 (53.0)	5.8	5.5	
Province category based on HDI*					0 < 0.001
Low^**^		2773 (36.2)	6.2	6.1	
Middle^***^		2553 (33.4)	5.1	4.7	
High^****^		2323 (30.4)	4.2	4.6	

Note: HDI^*^ is the human development index; Low^**^: Sistan and Baluchestan, Kurdistan, North Khorasan, South Khorasan, West Azerbaijan, Ardebil, Hormozgan, Zanjan, Hamadan, Golestan, Kerman provinces; Middle^***^: Razavi Khorasan, Lorestan, East Azerbaijan, Markazi, Kohgiluyeh Buyer Ahmad, Kermanshah, Chahar Mahall and Bakhtiari, Qazvin, Khuzestan, Gilan provinces; High^****^: Fars, Bushehr, Ilam, Qom, Semnan, Yazd, Mazandaran, Esfahan, Tehran, Alborz provinces

### Socioeconomic inequality in TEHB

**Fig. 2:**
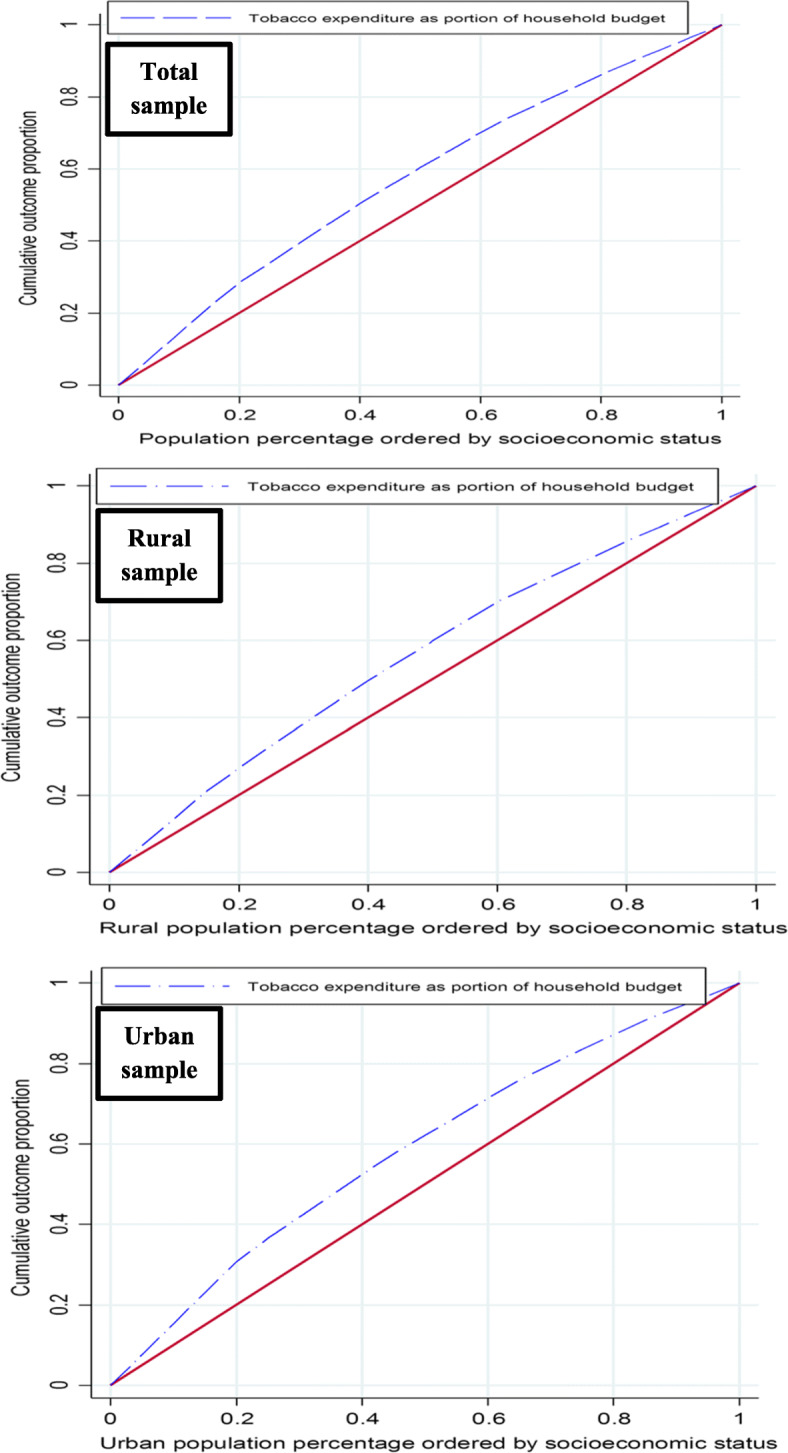
The concentration curve of tobacco expenditure as a proportion of household budget in Iran for the whole sample, rural and urban areas

The distribution of TEHB was different across the socioeconomic groups of households. The mean of TEHB for the poorest and the richest households was 7.6 and 3.6%, respectively (Table 1). The poorest households living in rural and urban areas spent 8.1 and 7.1% of their budgets on tobacco, while these the richest households spent 4.1 and 2.8%, respectively. Figure 2 shows the CCs of TEHB in Iran as well as in urban and rural areas. The CCs lie above the 45-degree line indicating concentration of TEHB among households with lower economic status. Table 2 presents the inequality estimates or the values of *C* at national level, for urban and rural areas and regions (provinces grouped by their development status). The *C* at national level is − 0.1423 (95% CI: − 0.1552 to − 0.1301), suggesting a pro-poor inequality in TEHB. In other words, TEHB was more concentrated among socioeconomically disadvantaged households in Iran. Similar results are found for urban (C = − 0.1707, 95% CI: − 0.1998 to − 0.1516) and rural (C = − 0.1314, 95% CI: − 0.1474 to − 0.1152) populations. Pro-poor inequalities in TEHB in provinces based on their HDI (the sign of *C* is negative and statistically significant). The magnitudes of observed inequality in three categories were different and higher for provinces with lower HDI (Table 2).

**Table 2:** The concentration indices for TEHB for whole sample, rural and urban areas and province category based on HDI in Iran

Sample	N	*C*	95% Confidence interval
*National level*	7649	− 0.1423	− 0.1552 to − 0.1301
*Urban/rural status*			
Urban area	3592	−0.1707	− 0.1898 to − 0.1516
Rural area	4057	− 0.1314	− 0.1474 to − 0.1152
*Province category based on HDI*			
Low^*^	2773	−0.1463	− 0.1662 to − 0.1264
Middle^**^	2553	− 0.1452	− 0.1651 to − 0.1252
High^***^	2323	− 0.1189	−0.1430 to − 0.0949

Note: TEHB is the tobacco expenditure as the share of the household budget; HDI is the human development index; C is the concentration index. Low*: Sistan and Baluchestan, Kurdistan, North Khorasan, South Khorasan, West Azerbaijan, Ardebil, Hormozgan, Zanjan, Hamadan, Golestan, Kerman provinces; Middle**: Razavi Khorasan, Lorestan, East Azerbaijan, Markazi, Kohgiluyeh Buyer Ahmad, Kermanshah, Chahar Mahall and Bakhtiari, Qazvin, Khuzestan, Gilan provinces; High***: Fars, Bushehr, Ilam, Qom, Semnan, Yazd, Mazandaran, Esfahan, Tehran, Alborz provinces

### Decomposing socioeconomic inequality in TEHB

Table 3 reports the results of the decomposition analysis of socioeconomic-related inequality in TEHB in Iran. There was a statistically significant and positive association between female-headed of household, age of household head, illiterate head of household, and residing in rural area, provinces with lower HDI and lower socioeconomic status with TEHB (column 2 in Table 3). The estimates of *C* of the explanatory variables indicate that households with higher proportion of males, age of household head, greater household size, and living in rural area were relatively rich. In contrary, female-headed household and illiterate head of household as well as provinces with lower development status were relatively poor (column 4 Table 3).

**Table 3:** Decomposition of socioeconomic inequality in TEHB^a^ among Iranian households, 2018

	Coefficient	Elasticity	*C*_*x*_^*b*^	Contribution to the *C*^*c*^		
				Contribution	%	Summed%
Demographic variables						
*Age of household head*	0.001^**^	1.023	0.004	0.004	−3.0	−3.0
*Gender of the household head*						
Male (ref.)						
Female	0.004^***^	0.009	−0.291	−0.003	1.9	1.9
*Proportion of males in the household*	−0.004^**^	−0.041	0.033	−0.001	0.9	0.9
Socioeconomic variables						
*Household size*						
Less than 4 (ref.)						
4 and above	−0.004^**^	−0.026	0.009	0.000	0.2	0.2
*Education status of the household head*						
Illiterate	0.009^*^	0.040	−0.193	−0.008	5.5	5.5
Literate (ref.)						
*Economic status of households*						
Poorest (ref.)						
Poor	−0.018^*^	−0.068	−0.407	0.030	−19.4	
Middle	−0.023^*^	−0.089	0.031	−0.003	1.9	
Rich	−0.034^*^	−0.129	0.445	−0.061	40.4	
Richest	−0.037^*^	−0.144	0.821	−0.128	83.2	106.1
Ecological variables						
*Geographical area*						
Urban (ref.)						
Rural	0.013^*^	0.117	0.055	0.006	−4.5	−4.5
*Province category based on HDI*^*d*^						
Low (ref.)						
Middle	−0.011^*^	−0.070	−0.027	0.002	−1.3	
High	−0.019^*^	−0.113	0.053	−0.006	4.2	2.9
Sum				**−0.156**		**109.9**
Residual				**−0.014**		**−9.9**
Total				**−0.142**		**100**

Note: TEHB is tobacco expenditure as the share of the household budget; C_x_ is the concentration index for explanatory variables; C is the concentration index for outcome variable and HDI is the human development index. ***Significant at 1%; **Significant at 5%; ^*^ Significant at 10%

According to column 4 in Table 3, he wealth status of the households was the most important factor in explaining inequality in TEHB. Besides wealth, gender of household’s head, education status of household head, proportion of males in the household, household size as well as development status of province contributed to the concentration of TEHB among the poor. For example, relative contribution of education of head of households to the overall inequality in TEHB in Iran was about 5.5%. This implies an equal distribution of education status of head of household, would lead a decrease 5.5% reduction socioeconomic-related inequality in TEHB. In contrast, other factors contributed to the concentration of TEHB among the better-off households were age of head of household (− 3.0%) and living in rural area (− 4.5%). If the age of head of households and geographic area were equally distributed among the households, socioeconomic inequality in TEHB would have been increased by 3 and 4.5%, respectively.

## Discussion

This study evaluated socioeconomic inequality in tobacco expenditure as the proportion of household budget (TEHB) as well as explained the main determinants of the observed inequality in Iran. The results show that about 20% of the households in Iran spent money from their budget on tobacco consumption, and these households spent about 5.2% of their monthly budget on buying tobacco-related products. This finding is in line with Turkish study, which found households spending about 8% of their monthly income on tobacco [4]. In Egypt, about 10% of household expenditures went to cigarettes or other forms of tobacco among lower-income households [31]. Our results also reveal that urban households in Iran allocated less of their budgets to spend on tobacco products compared to rural households. This finding corroborates a study in China where poorer households in urban and rural areas allocated 6.6 and 11.3% of their budgets to buy tobacco [33].

The most important finding of this study is that inequality in TEHB was pro-poor in Iran. In other words, the distribution of TEHB was more concentrated among the poorer households. Because of the prevalence of tobacco consumption was higher among poorer households and higher budget share of tobacco spending. The higher concentration of TEHB among the poorest could be explained by the fact that wealthier households have more money to purchase goods and services. Our results reflect the conclusion of the study from Hamadan city in Iran, where tobacco consumption was more prevalent among poorer adults [20]. The findings of this study are also consistent with the evidence from other developing countries. For example, tobacco use more prevalent among the poorest and poorer households were more affected by cigarette consumption compared to the wealthier households in Kenya and India [33]. In addition, wealth- and education-related inequalities in tobacco use were also found in 54 low-income countries and low-middle income countries [34]. In most of the countries, inequality of tobacco consumption was biased towards poorer and illiterate people.

This study also found that the extent of pro-poor inequality in TEHB was greater in urban areas and provinces with lower HDI. The reason is that TEHB significantly varied across the Iranian provinces. For example, TEHB among the households in Kohgiluyeh Buyer Ahmad was the lowest while it was the highest among the households from North Khorasan. Several factors could explain this variation in TEHB. For example, the development status of the province based on their HDI, proportion of households with tobacco expenditure more than zero (8.3% for north Khorasan vs 3.3% for that one) and sociocultural characteristics. The geographic location of the provinces could also be another factor behind this difference. For instance, North Khorasan is located the north-eastern region while Kohgiluyeh Buyer Ahmad is in the south-western region.

Findings from the decomposition analysis reveal that the wealth status of the households was the main contributor to the concentration of TEHB among the poorer households. This result is in line with findings from China, where economic status was found to be the main contributor to socioeconomic inequality tobacco consumption [24]. In Kenya, about 41% of the SES-related inequality in tobacco smoking was explained by the income of households [33]. Besides SES, education household heads and provincial development were the two most significant factors contributing to inequality in TEHB among the households with lower SES. Our empirical analysis suggests that if education status of head of the households and development status of provinces were equally distributed across socioeconomic groups, the SES-related inequality in TEHB in Iran would have been declined by 5.5 and 2.9%, respectively. This finding follows the results of the previous studies, which demonstrated a higher prevalence of smoking among low educated people [35].

The findings of our study have important implications for policymakers at national and sub-national levels in Iran. Although there are some studies on socioeconomic-related inequalities in the prevalence of tobacco consumption in Iran [20, 32, 36], this study presented the first empirical evidence on socioeconomic inequality in tobacco expenditure as the share of the households’ budget using nationally representative n data. Since tobacco expenditure was more concentrated among the poorest households, necessary policy interventions are needed to reduce tobacco use among them. Following WHO Framework Convention on Tobacco Control (FCTC) [37], one of the policies to reduce tobacco consumption could be increasing the price of tobacco products by imposing higher taxes on tobacco products mainly consumed by the poorest in Iran. Previous studies showed that low-income people change their tobacco consumption behaviour more due to price change [38, 39]. Therefore, increasing taxes on tobacco products would encourage poorer people to reduce or quit tobacco use and to spend less of their income on tobacco. This policy would help to protect socioeconomically disadvantage households from the financial burden of tobacco consumption. Besides raising taxes, another policy could be the banning of tobacco advertising in mass media and tobacco use in the public setting (https://www.who.int/news-room/detail/10-01-2017-tobacco-control-can-save-billions-of-dollars-and-millions-of-lives). In general, policies should be implemented to reduce the financial burden of tobacco consumption among the poorer households residing in rural areas and less developed provinces of Iran.

There are a few limitations that should be considered when interpreting the findings of this study. First, our findings are based on data from a cross-sectional survey, which limits the causal interpretation of the relationship between tobacco expenditure and its determinants. Second, self-reported data used in this study might underestimate or overestimate tobacco expenditures for the households. Besides, there could be a recall bias problem in our study. For example, poorer and low educated household heads might forget to report information related to tobacco expenditure. Third, other important determinants (e.g. occupation of the respondents) were not included in the analysis due to data limitation. The omission of such factors might have limited the decomposition of SES-related inequality in TEHB. Finally, the IHIES collected information at the household level. Therefore, we could not examine socioeconomic inequalities in tobacco expenditure at the individual level. Despite these limitations, our study contributed to the broader literature on SES-related inequalities in tobacco consumption in developing countries. Future studies should consider addressing above-mentioned limitations to offer robust empirical evidence in this area of research.

## Conclusion

To the best of our knowledge, our study is first of its kind to examine socioeconomic inequality in TEHB at national and subnational levels in Iran. We found evidence of SES-related inequality in cigarette expenditure in Iran, which was more concentrated among socioeconomically disadvantaged households. The wealth status of the households was the main contributing factor to the concentration of TEHB among the poorer households. The present study offers useful information for the policymakers to design and implement necessary interventions to reduce tobacco consumption among socioeconomically disadvantaged households. Further studies could consider examining the crowding-out effect of cigarette spending on the consumption of other goods in Iran.

## Supplementary information

**Additional file 1.** Proportion of households with positive tobacco expenditure in the past month by province in Iran, 2018. The proportion of households with tobacco expenditure more than zero in the last month was about 20%. These proportions for provinces based on their Human Development Index (HDI) were 18.7%, 22.9, and 18.8% (high, middle, and low, correspondingly).

## Data Availability

The data used in the study was extracted from the Household Income and Expenditure Surveys collected by the Iranian Statistical Center (ISC). The HIES is publicly available at https://www.amar.org.ir/english/Statistics-by-Topic/Household-Expenditure-and-Income#2220530-releases.

## Abbreviations

TEHB: Tobacco expenditure as the share of the household budget
HIES: Household income and expenditure survey
CC: Concentration curve
C: Concentration index
CI: Confidence interval
SES: Socioeconomic status
HDI: Human development index
OLS: Ordinary least square
