# Supplementary material for: Socioeconomic inequality in tobacco expenditure in Iran: a cross-sectional analysis at national and subnational levels
Source: BMC Public Health. 2020 Jun 29;20:1031. doi: 10.1186/s12889-020-09144-z (PMC7325296; doi:10.1186/s12889-020-09144-z)
Supplement: Supplementary file 1 — Additional file 1. Proportion of households with positive tobacco expenditure in the past month by province in Iran, 2018. The proportion of households with tobacco expenditure more than zero in the last month was about 20%. These proportions for provinces based on their Human Development Index (HDI) were 18.7%, 22.9, and 18.8% (high, middle, and low, correspondingly). [file 12889_2020_9144_MOESM1_ESM.docx]

**Supplementary file**

| Province | % |
| --- | --- |
| **Provinces with low HDI** |  |
| *Sistan and Baluchestan* | 7.1 |
| *Kurdistan* | 29.1 |
| *North Khorasan* | 15.4 |
| *South Khorasan* | 3.4 |
| *West Azerbaijan* | 26.9 |
| *Ardebil* | 29.7 |
| *Hormozgan* | 15.7 |
| *Zanjan* | 23.7 |
| *Hamadan* | 28.6 |
| *Golestan* | 16.1 |
| *Kerman* | 11.5 |
| **Provinces with middle HDI** |  |
| *Razavi Khorasan* | 15.0 |
| *Lorestan* | 22.1 |
| *East Azerbaijan* | 28.8 |
| *Markazi* | 23.7 |
| *Kohgiluyeh Buyer Ahmad* | 19.6 |
| *Kermanshah* | 21.4 |
| *Chahar Mahall and Bakhtiari* | 28.2 |
| *Qazvin* | 29.8 |
| *Khuzestan* | 21.0 |
| *Gilan* | 19.8 |
| **Provinces with high HDI** |  |
| *Fars* | 20.7 |
| *Bushehr* | 18.5 |
| *Ilam* | 11.2 |
| *Qom* | 22.4 |
| *Semnan* | 10.6 |
| *Yazd* | 18.7 |
| *Mazandaran* | 20.6 |
| *Esfahan* | 24.9 |
| *Tehran* | 17.3 |
| *Alborz* | 22.2 |
| ***Average in the provinces with low HDI*** | **18.8** |
| ***Average in the provinces with middle HDI*** | **22.9** |
| ***Average in the provinces with high HDI*** | **18.7** |
| ***Overall*** | **20.1** |

Table S1: Proportion of households with positive tobacco expenditure in the past month by province in Iran, 2018.

Note: HDI stands for human development index
